# Naturally Acquired Antibodies to Influenza A Virus in Fall-Migrating North American Mallards

**DOI:** 10.3390/vetsci9050214

**Published:** 2022-04-27

**Authors:** David E. Stallknecht, Alinde Fojtik, Deborah L. Carter, Jo Anne Crum-Bradley, Daniel R. Perez, Rebecca L. Poulson

**Affiliations:** 1 Southeastern Cooperative Wildlife Disease Study, Department of Population Health, College of Veterinary Medicine, University of Georgia, 589 D.W. Brooks Drive, Athens, GA 30602, USA; dstall@uga.edu (D.E.S.); afojtik@uga.edu (A.F.); dlcarter@uga.edu (D.L.C.); jcrum@uga.edu (J.A.C.-B.); 2 Poultry Diagnostic and Research Center, Department of Population Health, College of Veterinary Medicine, University of Georgia, 953 College Station Road, Athens, GA 30602, USA; dperez1@uga.edu

**Keywords:** *Anas platyrhynchos*, antibody, influenza A virus, population immunity

## Abstract

Although waterfowl are the primary reservoir for multiple subtypes of influenza A virus (IAV), our understanding of population immunity in naturally infected waterfowl is poorly understood. Population immunity may be an important driver of seasonal subtype predominance in waterfowl populations and may affect the potential for establishment of introduced IAV such as the Eurasian-like A/Goose/Guangdong/1/1996 lineage in these populations. Here, we examine the prevalence of naturally acquired antibodies to nucleoprotein (NP), hemagglutinin (H3, H4, H5), and neuraminidase (N1, N2, N6, N8) in early migrating mallards (*Anas platyrhynchos*) sampled in Northwest Minnesota during staging and early fall migration in September 2014, 2015, 2017, and 2018. Serologic results were compared to historic and contemporary virus isolation results from these same study sites. The prevalence of antibodies to NP ranged from 60.8–76.1% in hatch-year (HY) birds and from 86.0–92.7% in after-hatch-year (AHY, >1-year-old) mallards indicating a high level of previous infection with IAV early in the fall migration season. Neutralizing antibodies were detected against H3, H4, and H5 in all years as were antibodies to N1, N2, N6, and N8. A high proportion of NP seropositive ducks tested positive for antibodies to multiple HA and NA subtypes, and this was more common in the AHY age class. Antibody prevalence to the HA and NA subtypes included in this study were consistent with the predominance of H4N6 in these populations during all years and reflected a broadening of the antibody response with age. Additional work is needed to document the longevity of these immune responses, if and how they correlate with protection against IAV transmission, infection, and disease, and if, as detected in this study, they adequately describe the true extent of exposure to IAV or specific HA or NA subtypes.

## 1. Introduction

Waterfowl are naturally infected with multiple subtypes of influenza A virus (IAV) annually, but infections in temperate regions are seasonally variable as related to both IAV prevalence and subtype diversity [1]. In North American waterfowl, IAV prevalence in wild birds peaks in late summer and early fall, associated with premigration staging and early migration; this peak is associated with an annual influx of naïve hatch-year (HY) birds [2]. This same relationship with HY birds has been reported with IAV in waterfowl in Europe [3]. Infection prevalence in North American waterfowl decreases during late fall migration and wintering periods which is thought to be correlated with increased population immunity [4]. Seasonal patterns of subtype diversity also exist in North American duck populations with H3 and H4 subtypes predominating during late summer and early fall [1]. IAV subtype diversity in waterfowl increases during winter, and during spring migration when H7 and H10 subtypes may predominate [1,5,6].

Our current understanding of population immunity to IAV in naturally infected waterfowl is poorly understood and is complicated by extensive IAV subtype diversity and the repeated infections that occur within these populations. Although antibodies to IAV associated with nucleoprotein (NP) and hemagglutinin (HA) have been reported in wild ducks and other birds, it is not currently possible to translate these data to herd immunity; however, the prevalence of antibodies to IAV NP in waterfowl populations can be very high indicating a potentially general but measurable immune response to previous infections [3,7]. With subtype specific tests, such as hemagglutination inhibition (HI) and microneutralization (MN) assays, as applied to naturally infected wild birds, the sensitivity and specificity, the variation in antibody response associated with specific HA subtypes, the longevity of the detectable immune response, and the potential for cross-reactions associated with recurring infections with IAV representing multiple subtypes are not sufficiently defined and limit the translation of subtype-specific serologic results to population immunity. Antibodies to HA have been routinely detected in waterfowl, and as with NP, an antibody prevalence to individual HA subtypes as measured by HI and MN can be detected in a high proportion of tested waterfowl [8,9]. There has been limited work in ducks related to understanding or detecting antibodies to neuraminidase (NA), even though this is an important antigen associated with IAV response in both mice and humans and represents an independent correlate of protection [10,11]. Although much work is needed to fill these knowledge gaps, an understanding of herd immunity in naturally infected ducks is basic to understanding the natural history of IAV in wild waterfowl populations. Some applications of such knowledge include a better understanding of IAV subtype diversity patterns and the identification of potential immunologic barriers to the establishment of “new” viruses, such as highly pathogenic A/Goose/Guangdong/1/1996 H5 (GsGD-HP-H5) lineage IAV in wild bird reservoirs.

There are consistent IAV subtype patterns in North American ducks that might be attributable to immunological pressure. An example of this relates to the annual and seasonal predominance of H3 and H4 viruses in North American waterfowl, which has been reported at both local and flyway levels [1,12]. The prevalence of predominant subtype combinations associated with these HA subtypes (H3N8 and H4N6) can vary annually during these seasonal outbreaks, but both subtypes are usually present each year. Of the 7797 viruses reported from waterfowl in the Mississippi flyway from 1976 to 2015, H3N8 and H4N6 viruses represented 22% and 20% of total subtype diversity, respectively [1]. H3N6 and H4N8 subtypes also were commonly detected but represented only 4% and 5% of subtype diversity, respectively. On a more local scale in northwestern Minnesota, a similar pattern is reported from ducks sampled between 2007 and 2016 with H3N8 and H4N6 IAV predominating and cocirculating each year and representing 18% and 26% of total subtype diversity, respectively [12]. H3N6 and H4N8 viruses each represented 6% of detected subtype diversity during these same sampling periods. It is possible that the consistent predominance of H3N8 and H4N6 as opposed to H3N6 and H4N8 is associated with an increased replication fitness related to HA/NA functional balance [13]. However, population immunity directed at both the HA and NA of these dominant seasonal viruses (H3N8 and H4N6) may explain their subsequent reduction in prevalence during fall and winter and also act as a barrier to the success of reassortant subtype combinations (H3N6, H4N8) produced during coinfections with the predominant H3N8 and H4N6 subtypes.

Viruses of the GsGD-HP-H5 lineage have been consistently detected in wild waterfowl since 2002, and more recently have been detected annually in Europe [14]. In 2014, GsGD-HP-H5 was detected in domestic poultry and wild waterfowl in North America [15]. Although the progenitor of the 2014 outbreak was an H5N8 fully Eurasian GsGD-HP-H5 IAV, the most frequently detected subtype during the outbreak was a reassortant H5N2, with N2 originating from a North American lineage low-pathogenic (LP) IAV [16,17]. In Eurasia, multiple subtypes of GsGD-HP-H5 resulting from a reassortment with LP waterfowl lineages of IAV have also been reported including H5N1, H5N2, H5N3, H5N4, H5N6, and H5N8 [14]. Although the GsGD-HP-H5 viruses spread extensively in North America, they did not persist and were last detected during 2016 [18]. It is unknown why this virus did not establish in North America or why the H5N2 Eurasian/North American reassortant linage predominated, but both may have involved herd immunity pressure generated by previous infections with North American LP IAV [19]. Such immune pressure may relate to a combination of previous infections with LP H5 viruses, heterospecific immunity induced by exposure to other LP HA subtypes, or to immunity to NA [20,21,22,23,24].

In this study, we examine the prevalence of naturally acquired antibodies to NP, HA, and NA in early migrating mallards (*Anas platyrhynchos*) sampled as part of a long-term longitudinal IAV field study in Northwest Minnesota, USA. The objectives of this study were to determine: (1) if prevalence of antibodies to NP in mallards vary by age class and reflect the seasonal peaks in IAV infection prevalence annually observed during early migration; (2) if antibodies to H3, H4, and H5 in naturally infected mallards occur at levels reflecting the local occurrence and prevalence of viruses representing these HA subtypes; and (3) if antibodies to N1, N2, N6, and N8 can be detected in naturally infected mallards and reflect the local prevalence of viruses representing these NA subtypes. These objectives relate to building an understanding of the extent of measurable IAV immunity present in these early migrating mallards at a population level (antibodies to NP), understanding how population immunity could potential affect future subtype diversity of LP IAV (antibodies to H3, H4, N6, N8), and documenting the presence of potential population immunity barriers that could affect the outcome of an introduced GsGD-HP-H5 lineage IAV into North America (antibodies to H5, N1, N2, N6, N8).

## 2. Materials and Methods

In September 2014, 2015, 2017, and 2018, mallards were captured using rocket nets at three sites in northwestern Minnesota, USA; Roseau River Wildlife Management Area (Roseau County, 48.977714, −96.008911), Thief Lake Wildlife Management Area (Marshall County, 48.486903, −95.950603), and Agassiz National Wildlife Refuge (Marshall County, 48.300806, −95.980467). Waterfowl sampling at these sites was ongoing in 1989–1999 and in 2007–2018 [12,25,26]. All birds were classified as HY (<1-year-old) or after hatch year (AHY, >1-year-old) by plumage characteristics.

Cloacal/oropharyngeal (CL/OP) swabs for virus isolation were collected from all ducks and placed in a single vial containing 2 mL of chilled brain heart infusion broth (Becton Dickinson and Co., Sparks, ML, USA) supplemented with antimicrobials (penicillin G (1000 units/mL), streptomycin (1 mg/mL), kanamycin (0.5 mg/mL), gentamicin (0.25 mg/mL), and amphotericin B (0.025 mg/mL), Sigma-Aldrich, St Louis, MO, USA). Swabs were kept on ice packs for 24–36 h until shipped to the laboratory and placed in long-term storage at −70 °C until processed. Virus isolation, matrix rrt-PCR and H5 and H7 rrt-PCR, and subtyping were performed as previously described [12,27,28,29]. Any H5 or H7 positive samples were submitted to the National Veterinary Services Laboratory (Ames, IA, USA) for confirmation of pathogenicity and determination of subtype.

Blood (1–3 mL) was collected via jugular or brachial venipuncture from a subset of these ducks and sera was stored at −20 °C until testing. Sera were tested using a commercial blocking ELISA (IDEXX AI MultiS-Screen AB test, IDEXX Laboratories, Westbrook, ME, USA) for antibodies to the IAV NP according to the manufacturer’s instructions. Sera were considered positive for antibodies to IAV if the serum-sample-to-negative-control (S/N) absorbance value was less than 0.7 [30,31].

Antibodies to H3, H4, and H5 were tested using a microneutralization test (MN) at dilutions from 1:10–1:320 as previously described [32]. Two antigens, chosen based on subtype, were used for each HA and included: A/mallard/Minnesota/AI10-2593/2010(H3N8), A/mallard/Minnesota/AI10-3208/2008(H4N6), A/mallard/Minnesota/AI11-3933/2011(H5N1), RG-A/mallard/Minnesota/Sg-000169-CRIP102_RGJCB1/2007 (H3N8), RG-A/mallard/Minnesota/AI10-3208-CRIP102_RGJCB3/2010 (H4N6), and RG-A/mallard/Minnesota/A108-5478-CRIP102_RGJCB5/2008 (H5N2). A titer of 10 to either HA antigen was regarded as seropositive.

Antibodies to N1, N2, N6, and N8 were tested using an enzyme-linked lectin assay (ELLA). Antigens were propagated in ECE as previously described [27]; one antigen was used for each NA subtype and included: A/ruddy turnstone/New Jersey/AI13-2948/2013(H10N1), A/ruddy turnstone/New Jersey/AI13-2891/2013(H10N2), A/mallard/Minnesota/AI10-2502/2010(H10N6), and A/ruddy turnstone/New Jersey/AI13-2208/2013(H10N8). To determine the appropriate amount of each antigen to use in ELLA, viruses were diluted from 1:10–1:10,240, and allowed to incubate on fetuin-coated plates at 37 °C under CO_2_ for 16–18 h. After incubation, plates were washed with PBS-Tween, and a lectin (peanut agglutinin-horseradish peroxidase (PNA-HRPO); Sigma-Aldrich Inc., St. Louis, MO, USA) was added to each well, followed by a chromogenic peroxidase substrate (O-phenylenediamine dihydrochloride; Sigma Aldrich) prepared in citrate buffer (Sigma Aldrich) as has been previously described [33,34]. Absorbance at 492 nm for 0.1 s was measured using an EMax Plus microplate reader (Molecular Devices, San Jose, CA, USA). The optimal quantity of antigen was determined to be the dilution that yielded 90–95% of maximum optical density (OD). Antigens were screened against ferret-derived antisera for each NA to determine the optimal concentration to use as a control in ELLA assays and to limit cross-reactivity. Test sera were heat-inactivated at 57 °C for 45 min and then screened against each antigen (N1, N2, N6, N8). Briefly, sera were diluted 1:10 in DPBS supplemented with 1% bovine serum albumin and 0.5% Tween 20 and transferred (50 μ L) to individual wells of a washed fetuin coated plate. To each well, 50 uL of diluted antigen was added. Controls included wells that contained virus only (four per plate), background only (eight per plate), and appropriately matched ferret anti-sera to respective NA (two per plate). Plates were incubated at 37 °C under CO_2_ for 16–18 h, and the remaining steps were carried out as described above. For each antigen, sera that showed >50% inhibition were then titrated two-fold from 1:10–1:640. For the screens and titrations to be considered valid, background values on each plate were less than 10% of the virus-only control, matched ferret anti-sera yielded >50% inhibition, and virus only controls were 90–95% of maximum OD with less than 10% difference between each replicate on the plate. If sera did not show inhibition of antigen at a starting dilution of 1:10, they were considered antibody negative to that antigen; if inhibition was observed, the positive titer was recorded as the highest dilution at which ≥50% inhibition of maximum optical signal was observed.

Differences between antibody prevalence estimates were tested by Mantel–Haenszel chi-square tests using Epi InfoTM for Windows software.

## 3. Results

Waterfowl sampling: CL/OP swabs were collected from 532, 586, 794, and 273 mallards during 2014, 2015, 2017, and 2018, respectively (Appendix A Table A1). Blood was collected from a subset of these same ducks with 111, 435, 149, and 92 sera tested during 2014, 2015, 2017, and 2018, respectively (Table 1).

**Figure 1:**
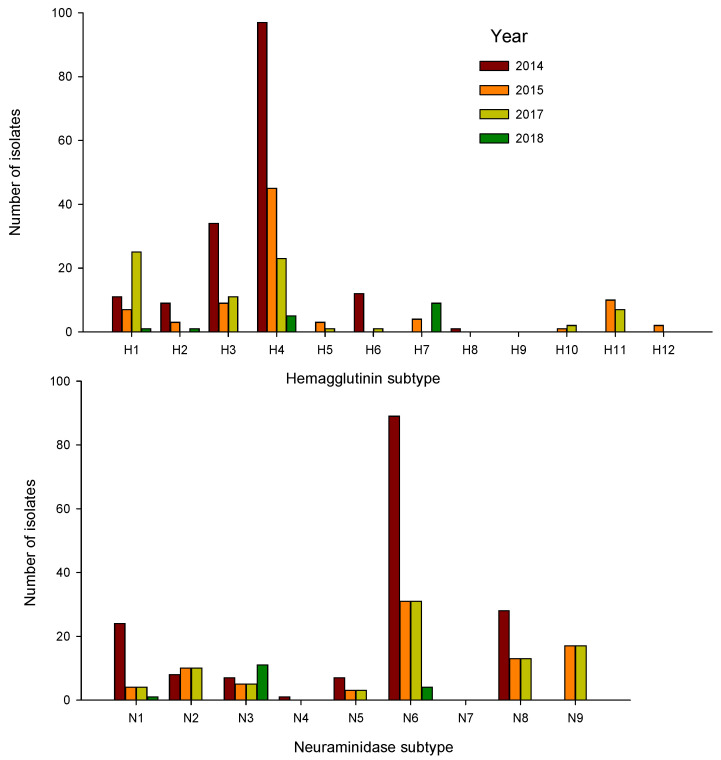
Influenza A virus hemagglutinin and neuraminidase subtypes detected in wild mallards sampled in Northwest Minnesota, USA during 2014, 2015, 2017, and 2018.

Virus isolation: A total of 350 IAVs were isolated with HA/NA subtype combinations determined for 329 (Table 2; Appendix A Table A1). Prevalence of infection varied by year from 31.6% in 2014 to 8.8% in 2017. Forty HA/NA subtype combinations were detected with HA 1–8, 10–12, and all avian NA subtypes (N1-N9) represented (Table 2). Predominant HA subtypes included H1, H3, and H4; 80.9% of total isolates were represented by these three HA subtypes (Figure 1). Predominant NA subtypes included N8, N6, and N1, which together represented 76.6% of all NA subtype diversity (Figure 1). The five most common subtype combinations included H1N1, H3N6, H3N8, H4N6, and H4N8, which together made up 67% of total isolates while only representing 12% of HA/NA subtype combination diversity. Of these, the H1, H4, N1, N6, and N8 subtypes were detected in all years; H3 was detected in 2014, 2015, and 2017 (Figure 1). Only four LP H5 IAVs were detected; one H5N2 and two H5N9 in 2015 and one H5N7 in 2017.

NP antibody prevalence: A high antibody prevalence was detected in both HY and AHY birds (Table 2). Depending on the year, NP antibody prevalence ranged from 50.7% to 76.1% in HY and from 86.0% to 92.7% in AHY mallards. Overall, antibody prevalence estimates were significantly higher (*p* ≤ 0.001) for AHY (90.1%) than HY (59.3%) mallards. For individual years, antibody prevalence was significantly higher in AHY than HY birds in 2015 (*p* ≤ 0.001) and 2018 (*p* = 0.001); although a significant difference could not be detected, antibody prevalence estimates for AHY ducks did exceed those for HY birds during 2014 and 2017.

**Figure 2:**
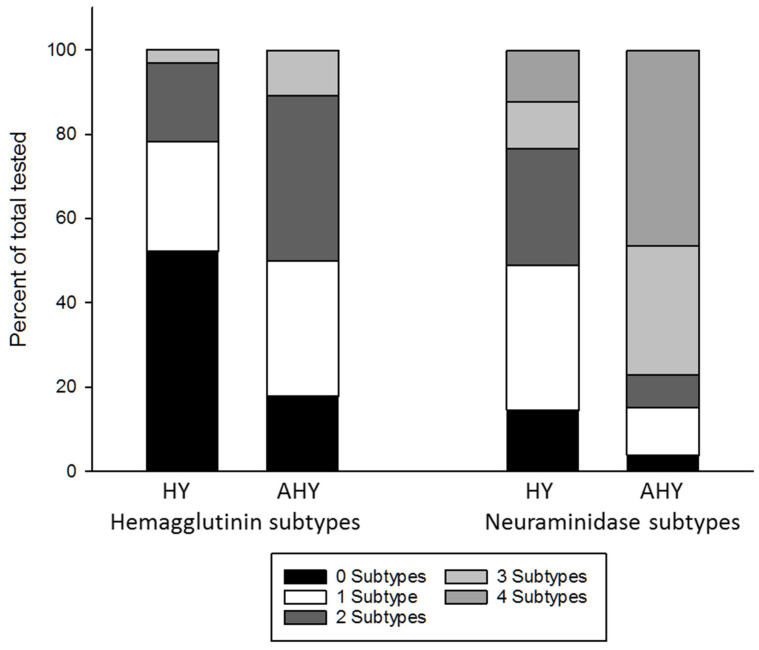
Percentage of tested hatch-year (HY) and after-hatch-year (AHY) influenza A virus nucleoprotein antibody-positive mallards testing positive to 0, 1, 2, or 3 of 3 hemagglutinin (H3, H4, H5) and 0, 1, 2, 3, or 4 of 4 neuraminidase (N1, N2, N6, N8) subtypes.

HA and NA antibody prevalence: Neutralizing antibodies were detected against H3, H4, and H5 in all years (Table 3). For the total sample, significant differences in antibody prevalence between HY and AHY birds were detected for H3 (*p* = 0.02) and H5 (*p* ≤ 0.001). For H4, a significant difference in antibody prevalence observed in HY (30%) and AHY (36%) birds was not detected. Antibodies to N1, N2, N6, and N8 were detected in birds in all years (Table 3). For the total sample, significant differences in antibody prevalence between HY and AHY birds were detected for N1 (*p* ≤ 0.001), N2 (*p* ≤ 0.001), and N8 (*p* ≤ 0.001). For N6, significant differences between antibody prevalence estimates for HY (78%) and AHY (83%) were not detected. A high proportion of NP seropositive ducks tested positive for antibodies to multiple HA and NA subtypes, and this was more common in the AHY age class (Figure 2). For HY and AHY birds, 22% and 50% (*p* = 0.004) tested seropositive for more than one of the three HA subtypes included in this study, respectively. For HY and AHY birds, 51% and 85% (*p* = 0.002) tested seropositive for more than one NA subtypes, respectively.

Of the H3 seropositive mallards, 94% and 91% percent tested positive to N6 and N8, respectively. For H4 seropositive birds 100% and 63% percent tested seropositive to N6 and N8, respectively.

## 4. Discussion

The observed prevalence of IAV infection and subtype diversity was consistent with past reports from northwestern Minnesota and from the Mississippi flyway, with H3, H4, N6, and N8 subtypes most commonly detected [1,12,25,26]. During the years included in this study, H4 subtypes dominated every year with H4N6 representing the most common subtype combination in all years. Although sera were not available for 2016, this same subtype pattern was observed in that year [12]. The H4 predominance for five consecutive years is unusual as shifts in the relative frequency of detected H3 and H4 IAV between years are more expected [1,12]. The low prevalence and sporadic detection of H5 IAV at this site during September also is consistent with previous studies at these same study sites [25,26]. It is important to note, however, that the virus isolation results reported in the present study reflect only a portion of the overall premigration/early migration IAV transmission period, which begins in late July. Subtype diversity at these sites can change during this time especially related to the detection of less predominant subtypes such as H5 [26]. In a study in California, LP H5 IAVs were commonly detected in breeding mallards sampled in July and August [35].

NP antibody prevalence estimates were high in both HY and AHY birds, indicating that a large proportion of both age classes had been previously infected with one or more IAVs prior to sampling in early September. In all years, NP antibody prevalence was highest in AHY birds, but significant differences were only detected during 2015 and 2018. With HY birds, during 2014 and 2017, over 75% and 76% of birds were seropositive to NP, respectively. This may indicate increased transmission of IAV during these years or may relate to the timing of peak transmission in relation to sampling; both can vary between years [26].

Although most IAV isolations were represented by H4 subtypes, overall HA antibody prevalence estimates in HY birds were not significantly higher than prevalence estimates for H3 or H5. The relative frequencies of antibodies to H3 and H4, however, did reflect subtype diversity related to the virus isolation data. These low prevalence estimates may reflect the beginning of the annual IAV transmission cycle in ducks, but they may also reflect low test sensitivity and an underestimate of actual exposure. This possibility is supported by the low neutralization titers observed in both the H3 and H4 MN results (Supplemental Table S1). Even though antibody prevalence was generally higher in AHY birds, low titers were also observed in this age class. Antibody prevalence to H5 in HY birds ranged from 5% to 38% between years (Table 3). The elevated prevalence of antibodies to H5 in HY birds during 2015 coincided with the detection of H5 IAV that year.

Overall antibody prevalence estimates for HY and AHY birds were significantly different for H3 and H5 but not H4. Historically in the Mississippi flyway, H5 IAVs are most often detected in ducks in fall (September–November) rather than summer (June–August), while H3 and H4 IAVs generally are very common during summer [1]. We do not know how long neutralizing antibodies can be detected in naturally infected ducks, but if less than one year, this seasonal pattern could partially explain these differences. Loss of neutralizing antibodies to various IAV subtypes has been previously documented in naturally infected mallards sampled through winter and occurs more frequently in HY birds [32]. The detection of age-related differences in H3 but not H4 antibody prevalence may be associated with the predominance of the H4 subtype and the increased probability of recent infections in HY birds at this study site during all years.

Although observed antibody prevalence estimates to N2, N6, and N8 were higher than those observed for the HA subtypes, general patterns were identical. The relative differences in antibody prevalence for these NA subtypes mirrored the virus isolation data with the highest antibody prevalence observed for N6. Differences between HY and AHY age classes were detected with N1, N2, and N8 but not with N6. This parallels results for differences in antibody prevalence observed for HA and probably relates to predominance of H4N6 observed in all years of this study. Although we did not have sufficient data to identify a predominant H5 subtype during these four years, H5N2 has been the most detected subtype combination at these sites and from ducks in the Mississippi flyway [1,26].

The NP antibody data indicate that a relatively high proportion of HY ducks (51–56%) are infected with IAV early in the migratory period. In AHY birds, 83–93% were NP-antibody-positive. The HA and NA antibody data also suggest that this immunity reflects exposure to multiple subtypes and broadens with age. With HA, this same relationship with age is reported in mute swans (*Cygnus olor*) and is more pronounced with a greater range of age classes represented in that study [8].

Because all four years of this study were predominated by H4N6, our results do not give a clear indication of the potential for herd immunity to affect reassortment between seasonally cocirculating IAVs such as H3N8 and H4N6. The high prevalence of N6 antibodies likely associated with H4N6 predominance, however, suggests that significant immune pressure may exist for subsequent H3N6 infections. With regard to potential population immunity barriers that could affect the outcome of an introduced GsGD-HP-H5 linage virus into North America, it has been clearly demonstrated that prior infection with North American LP H5 IAV can affect the outcome of subsequent infections with GsGD-HP-H5 lineage IAV [20,21]. This outcome may range from complete protection against infection, reduced viral shedding, an increase in the infective dose, or to reduced morbidity and mortality. These outcomes could result in a lower probability of subsequent transmission; however, decreased morbidity and mortality also may result in increased geographic dispersal of the virus. Our results indicate that by mid-September antibodies to NP are present in a large proportion of ducks. Antibodies to H5 and NA subtypes (N1, N2, N6, N8) that have been associated with GsGD-HP-H5 lineage IAV also can be detected. These results document existing herd immunity to LP IAV early in the migration cycle that may precede a later introduction of an IAV. Although it is not clear when in the migration cycle such introductions are likely to occur throughout North America, the 2014 and most recent 2021 detections of GsGd-HP-H5 IAV in North America occurred in late fall and winter [15]. A similar seasonal timing has been reported for the detection of GsGD-HP-H5 IAV in Europe [14,36]. If prior exposure to LP IAV provides any immune pressure relative to such an introduction, the timing of these LP infections and the prevalence of antibodies to specific HA and NA subtypes during that year may be relevant to the potential trajectory of an introduced GsGD-HP-H5 virus.

The observed differences in antibody prevalence between HY and AHY birds reinforces the need to consider the effect of the population age-structure on such immune pressure. Duck populations can have a high annual recruitment rate that varies annually related to breeding success. The proportional population of HY and AHY birds as well as their immune status needs to be considered in any assessment of population immunity and this may be especially true in years with a high reproductive success.

In this study we provide evidence that antibodies to NP, HA, and NA, resulting from natural IAV infections in wild mallards, do reflect the local IAV exposure history as indicated by virus isolation. However, this is a small step in attempting to translate serologic results to populations immunity. Questions remain regarding the longevity of these immune responses, if and how they correlate with protection, transmission, infection, and disease, and if, as detected in this study, they adequately describe the true extent of exposure to IAV or specific HA, or NA subtypes.

## 5. Conclusions

Population immunity is an important driver of seasonal IAV subtype diversity and predominance in naturally exposed wild birds and likely affects the successful establishment of introduced IAVs of concern. However, the longevity and specificity of such immune responses are largely unknown, as are the roles these responses might play in IAV transmission, infection, and disease in wild bird populations. Here, we examined the prevalence of naturally acquired antibodies in mallards in northwestern Minnesota over four years and found that they were reflective of the local IAV exposure history. Research questions aimed at unravelling the complexity of immunity to IAV in wild birds, and continued refinement of serological tools used to answer these questions, are critical to better understand the natural history of IAV in wild avian populations.

## Figures and Tables

**Table 1:** Prevalence of antibodies to influenza A virus in sera collected from wild mallards in Northwest Minnesota, USA, by year and age class.

Year	Age Class ^1^	Number Tested	Number Positive	Percent Positive (95% Confidence Limits)
2014	HY	89	67	75% (0.65–0.84)
	AHY	22	20	91% (0.71–0.99)
2015	HY	353	179	51% (0.45–0.56)
	AHY	82	76	93% (0.85–0.97)
2017	HY	92	70	76% (0.66–0.84)
	AHY	57	49	86% (0.74–0.94)
2018	HY	51	31	61% (0.46–0.74)
	AHY	41	37	90% (0.77–0.97)
Total	HY	585	347	59% (0.55–0.63)
	AHY	202	182	90% (0.85–0.93)

^1^ Hatch year (HY), after-hatch year (AHY).

**Table 2:** Influenza A virus subtype diversity detected in wild mallards sampled in Northwest Minnesota, USA in 2014, 2015, 2017, and 2018, totaled across all years.

Neuraminidase Subtype	Hemagglutinin Subtype												Total
	H1	H2	H3	H4	H5	H6	H7	H8	H9	H10	H11	H12	
N1	34		6	6		9					2		57
N2	1		2	15	1						1		20
N3	1	7		2			12						22
N4								1					1
N5	2	4	1	1		1						1	10
N6	1	1	14	124		2							142
N7					1					1			2
N8	2		29	20		1					1		53
N9	3	1	1	1	2					2	12		22
Total	44	13	53	169	4	13	12	1	0	3	16	1	329

**Table 3:** Antibodies to hemagglutinin (microneutralization) and neuraminidase (ELLA) in sera collected from wild mallards in Northwest Minnesota, USA, by year and age class.

Year	Age Class ^1^	*n*	Microneutralization (Number Positive (% Positive))			ELLA (Number Positive (% Positive))			
			H3	H4	H5	N1	N2	N6	N8
2014 ^2^	HY	21	2 (10%)	4 (20%)	1 (5%)	3 (16%)	3 (14%)	14 (67%)	4 (20%)
	AHY	5	0	2 (40%)	0	0	1 (20%)	4 (80%)	1 (20%)
2015	HY	26	9 (35%)	9 (35%)	10 (38%)	4 (15%)	8 (31%)	19 (73%)	12 (46%)
	AHY	5	2 (40%)	1 (20%)	5 (100%)	2 (40%)	5 (100%)	3 (60%)	5 (100%)
2017	HY	24	5 (21%)	9 (38%)	6 (25%)	6 (25%)	7 (29%)	19 (79%)	12 (50%)
	AHY	4	1 (25%)	1 (25%)	3 (75%)	3 (75%)	4 (100%)	3 (75%)	3 (75%)
2018	HY	21	4 (19%)	6 (29%)	5 (24%)	5 (24%)	10 (48%)	20 (95%)	10 (48%)
	AHY	14	10 (71%)	6 (43%)	9 (64%)	9 (64%)	12 (86%)	13 (93%)	13 (93%)
Total	HY	92	20 (22%)	28 (30%)	22 (24%)	18 (20%)	28 (30%)	72 (78%)	38 (41%)
	AHY	28	13 (46%)	10 (36%)	17 (61%)	14 (54%)	22 (79%)	23 (83%)	22 (79%)

^1^ Hatch year (HY), after-hatch year (AHY). ^2^ Sample size for 2014 N1: HY = 19; AHY = 3. Total sample size N1: HY = 90; AHY = 26.

## Data Availability

The data presented in this study are available in Appendix A Table A1 and Supplemental Table S1 of this article.

## Supplementary Materials

The following supporting information can be downloaded at: https://www.mdpi.com/article/10.3390/vetsci9050214/s1, Supplemental Table S1: Mallards tested for antibodies to influenza A virus nucleoprotein hemagglutinin and neuraminidase.

## Appendix A

**Table A1:** Virus isolation results from Mallards sampled in Northwest Minnesota.

Year	Dates Sampled	Number Tested	Number IAV Positive	Percent Positive (95% Confidence Limits)	Subtypes Isolated (Subtype (Number))
2014	28 August 2014–13 September 2014	532	168	31.6% (0.28–0.36)	H1N1(7), H1N3(1), H1N5(1), H1N6(1), H1N8(1), H2N3(5), H2N5(3), H2N6(1), H3N1(5), H3N2(2), H3N5(1), H3N6(6), H3N8(19), H3Nx(1), H4Nx(1), H4N1(4), H4N2(6), H4N3(1), H4N5(1), H4N6(76), H4N8(9), H6N1(8), H6N5(1), H6N6(2), H6N8(1), H8N4(1), HxN5(1), HxN6(1), mixed ^a^ (1)
2015	11 September 2015–23 September 2015	586	87	14.9% (0.12–0.18)	H1N1(2), H1N2(1), H1N5(1), H1N9(3), H2N3(1), H2N5(1), H2N9(1), H3N6(1), H3N8(7), H3N9(1), H4N1(1), H4N2(7), H4N6(29), H4N8(6), H4N9(1), H5N2(1), H5N9(2), H7Nx(2), H7N3(3), H10N9(1), H11N1(1), H11N2(1), H11N9(8), H12N5(1), HxN6(1), HxN9(2), mixed(1)
2017	6 September 2017–21 September 2017	794	70	8.8% (0.07–0.11)	H1N1(24), H1N8(1), H3N1(1), H3N6(7), H3N8(3), H4N1(1), H4N2(2), H4N6(15), H4N8(5), H5N7(1), H6N1(1), H10N7(1), H10N9(1), H11N1(1), H11N8(1), H11N9(4), mixed(1)
2018	30 August 2018–20 September 2018	273	25	9.2% (0.06–0.13)	H1N1(1), H2N3(1), H4N3(1), H4N6(4), H4Nx(2), H7N3(9), HxN6(1), HxNx ^b^ (6)

^a^ mixed: HA and/or NA subtypes were determined to be mixed. ^b^ HxNx: neither HA nor NA subtypes were determined, but sample was an IAV isolate.
